# Supplementary material for: Antagonism of PP2A is an independent and conserved function of HIV-1 Vif and causes cell cycle arrest
Source: eLife. 2020 Apr 15;9:e53036. doi: 10.7554/eLife.53036 (PMC7920553; doi:10.7554/eLife.53036)
Supplement: Supplementary file 1. — Sequences of PCR primers for Vif mutant library construction, codon-optimised Vif variants synthesised as gBlocks, Vif coding sequences in HIV-AFMACS viruses, the C-terminal 4xHA-tagged APOBEC3F coding sequence in pHRSIN-S-W-pGK puro, oligonucleotides for RNAi and primers for qRT-PCR. [file elife-53036-supp1.docx]

**DNA and RNA sequences**

**PCR primers for Vif mutant library construction**

| **Mutant** | **Primer** | **Sequence^1^** |
| --- | --- | --- |
| R15A | 15A_Fwd | TGGCAGGTGGACGCCATGCGCATTAACACC |
|  | 15A_Rvs | GTTAATGCGCATGGCGTCCACCTGCCACAC |
| K26A | 26A_Fwd | AAGCGCCTGGTGGCCCACCACATGTACATT |
|  | 26A_Rvs | GTACATGTGGTGGGCCACCAGGCGCTTCCA |
| Y30A | 30A_Fwd | AAGCACCACATGGCCATTAGCCGCAAAGCT |
|  | 30A_Rvs | TTTGCGGCTAATGGCCATGTGGTGCTTCAC |
| I31A | 31A_Fwd | CACCACATGTACGCCAGCCGCAAAGCTAAG |
|  | 31A_Rvs | AGCTTTGCGGCTGGCGTACATGTGGTGCTT |
| I31D | 31D_Fwd | CACCACATGTACGACAGCCGCAAAGCTAAG |
|  | 31D_Rvs | AGCTTTGCGGCTGTCGTACATGTGGTGCTT |
| RK33/34A | 33/34A_Fwd | ATGTACATTAGCGCCGCCGCTAAGGACTGGTTCTAC |
|  | 33/34A_Rvs | GAACCAGTCCTTAGCGGCGGCGCTAATGTACATGTG |
| RK33/34EE | 33/34EE_Fwd | ATGTACATTAGCGAGGAGGCTAAGGACTGGTTC |
|  | 33/34EE_Rvs | CCAGTCCTTAGCCTCCTCGCTAATGTACATGTG |
| K36A | 36A_Fwd | AGCCGCAAAGCTGCCGACTGGTTCTACCGC |
|  | 36A_Rvs | GTAGAACCAGTCGGCAGCTTTGCGGCTAAT |
| F39A | 39A_Fwd | GCTAAGGACTGGGCCTACCGCCACCACTAC |
|  | 39A_Rvs | GTGGTGGCGGTAGGCCCAGTCCTTAGCTTT |
| Y44A | 44A_Fwd | TACCGCCACCACGCCGAGAGCACCAACCCC |
|  | 44A_Rvs | GTTGGTGCTCTCGGCGTGGTGGCGGTAGAA |
| D61A | 61A_Fwd | ATTCCCCTGGGCGCCGCCAAGCTGGTGATT |
|  | 61A_Rvs | CACCAGCTTGGCGGCGCCCAGGGGAATGTG |
| K63A | 63A_Fwd | CTGGGCGACGCCGCCCTGGTGATTACGACC |
|  | 63A_Rvs | GGTCGTAATCACCAGGGCGGCGTCGCCCAG |
| W70R | 70R_Fwd | ATTACGACCTACCGGGGCCTGCACACCGGC |
|  | 70R_Rvs | GGTGTGCAGGCCCCGGTAGGTCGTAATCAC |
| E76R | 76R_Fwd | CTGCACACCGGCCGGCGCGACTGGCACCTG |
|  | 76R_Rvs | GTGCCAGTCGCGCCGGCCGGTGTGCAGGCC |
| W79A | 79A_Fwd | GGCGAGCGCGACGCCCACCTGGGCCAGGGC |
|  | 79A_Rvs | CTGGCCCAGGTGGGCGTCGCGCTCGCCGGT |
| R93A | 93A_Fwd | TGGAGGAAAAAGGCCTATAGCACACAAGTA |
|  | 93A_Rvs | TTGTGTGCTATAGGCCTTTTTCCTCCATTC |
| S116A | 116A_Fwd | TTTGATTGTTTTGCCGAATCTGCTATAAGA |
|  | 116A_Rvs | TATAGCAGATTCGGCAAAACAATCAAAATA |

1. Mutagenised codons shaded in grey.

**PCR primers for Vif mutant library construction (cont.)**

| **Mutant** | **Primer** | **Sequence^1^** |
| --- | --- | --- |
| E117A | 117A_Fwd | GATTGTTTTTCAGCCTCTGCTATAAGAAAT |
|  | 117A_Rvs | TCTTATAGCAGAGGCTGAAAAACAATCAAA |
| S118A | 118A_Fwd | TGTTTTTCAGAAGCCGCTATAAGAAATACC |
|  | 118A_Rvs | ATTTCTTATAGCGGCTTCTGAAAAACAATC |
| R121A | 121A_Fwd | GAATCTGCTATAGCCAATACCATATTAGGA |
|  | 121A_Rvs | TAATATGGTATTGGCTATAGCAGATTCTGA |
| N122A | 122A_Fwd | TCTGCTATAAGAGCCACCATATTAGGACGT |
|  | 122A_Rvs | TCCTAATATGGTGGCTCTTATAGCAGATTC |
| R127A | 127A_Fwd | ACCATATTAGGAGCCATAGTTAGTCCTAGG |
|  | 127A_Rvs | AGGACTAACTATGGCTCCTAATATGGTATT |
| R127E | 127E_Fwd | ACCATATTAGGAGAGATAGTTAGTCCTAGG |
|  | 127E_Rvs | AGGACTAACTATCTCTCCTAATATGGTATT |
| I128A | 128A_Fwd | ATATTAGGACGTGCCGTTAGTCCTAGGTGT |
|  | 128A_Rvs | CCTAGGACTAACGGCACGTCCTAATATGGT |
| I128D | 128D_Fwd | ATATTAGGACGTGACGTTAGTCCTAGGTGT |
|  | 128D_Rvs | CCTAGGACTAACGTCACGTCCTAATATGGT |
| S130A | 130A_Fwd | GGACGTATAGTTGCCCCTAGGTGTGAATAT |
|  | 130A_Rvs | TTCACACCTAGGGGCAACTATACGTCCTAA |
| S130E | 130E_Fwd | GGACGTATAGTTGAGCCTAGGTGTGAATAT |
|  | 130E_Rvs | TTCACACCTAGGCTCAACTATACGTCCTAA |
| P131A | 131A_Fwd | CGTATAGTTAGTGCCAGGTGTGAATATCAA |
|  | 131A_Rvs | ATATTCACACCTGGCACTAACTATACGTCC |
| R132D | 132D_Fwd | ATAGTTAGTCCTGACTGTGAATATCAAGCA |
|  | 132D_Rvs | TTGATATTCACAGTCAGGACTAACTATACG |
| L153A | 153A_Fwd | GCACTAGCAGCAGCCATAAAACCAAAACAG |
|  | 153A_Rvs | TTTTGGTTTTATGGCTGCTGCTAGTGCCAA |
| I154A | 154A_Fwd | CTAGCAGCATTAGCCAAACCAAAACAGATA |
|  | 154A_Rvs | CTGTTTTGGTTTGGCTAATGCTGCTAGTGC |
| K155A | 155A_Fwd | GCAGCATTAATAGCCCCAAAACAGATAAAG |
|  | 155A_Rvs | TATCTGTTTTGGGGCTATTAATGCTGCTAG |
| K157E | 157E_Fwd | TTAATAAAACCAGAGCAGATAAAGCCACCT |
|  | 157E_Rvs | TGGCTTTATCTGCTCTGGTTTTATTAATGC |
| K160E | 160E_Fwd | CCAAAACAGATAGAGCCACCTTTGCCTAGT |
|  | 160E_Rvs | AGGCAAAGGTGGCTCTATCTGTTTTGGTTT |
| N/A | seq | gaagcaggctggagacgtggag |
|  | Vif_Fwd | CattcacaggtgcagctcgaggggtcagggATGGAGAACCGG |
|  | Vif_Rvs | AattttgtaatccagaggttgattggtaccCTAGTGTCCATTCAT |

1. Mutagenised codons shaded in grey.

**Codon-optimised Vif variants synthesised as gBlocks**

| **Vif variant** | **Sequence^1^** |
| --- | --- |
| NL4-3 | cattcacaggtgcagctcgaggggtcagggATGGAGAACCGGTGGCAGGTGATGATTGTGTGGCAGGTGGACCGCATGCGCATTAACACCTGGAAGCGCCTGGTGAAGCACCACATGTACATTAGCCGCAAAGCTAAGGACTGGTTCTACCGCCACCACTACGAGAGCACCAACCCCAAGATTAGCAGCGAGGTGCACATTCCCCTGGGCGACGCCAAGCTGGTGATTACGACCTACTGGGGCCTGCACACCGGCGAGCGCGACTGGCACCTGGGCCAGGGCGTCTCCATAGAATGGAGGAAAAAGAGATATAGCACACAAGTAGACCCTGACCTAGCAGACCAACTAATTCATCTGCACTATTTTGATTGTTTTTCAGAATCTGCTATAAGAAATACCATATTAGGACGTATAGTTAGTCCTAGGTGTGAATATCAAGCAGGACATAACAAGGTAGGATCTCTACAGTACTTGGCACTAGCAGCATTAATAAAACCAAAACAGATAAAGCCACCTTTGCCTAGTGTTAGGAAACTGACAGAGGACAGATGGAACAAGCCCCAGAAGACCAAGGGCCACAGAGGGAGCCATACAATGAATGGACACTAGggtaccaatcaacctctggattacaaaatt |
| HXB2 | cattcacaggtgcagctcgaggggtcagggATGGAAAACAGATGGCAGGTTATGATCGTGTGGCAAGTAGATCGCATGAGAATTAGAACTTGGAAGTCATTGGTGAAGCATCACATGTACGTGAGCGGAAAGGCGCGAGGATGGTTCTATAGACATCATTACGAGTCCCCGCATCCACGGATCAGCTCAGAGGTCCATATCCCACTTGGCGATGCACGGCTTGTGATTACAACTTATTGGGGTCTTCACACTGGGGAACGGGACTGGCATCTGGGCCAGGGAGTGAGTATTGAGTGGAGAAAAAAAAGGTATAGCACTCAGGTAGACCCCGAACTCGCGGATCAACTGATACACTTGTATTACTTCGACTGCTTCTCTGACAGCGCGATCCGAAAGGCATTGCTCGGCCATATCGTTAGTCCGCGCTGTGAGTATCAAGCCGGGCATAACAAGGTCGGTTCTCTTCAATATCTGGCGCTCGCAGCGCTTATCACCCCGAAAAAGATTAAACCTCCTTTGCCATCAGTTACCAAGCTGACCGAGGACAGGTGGAACAAACCCCAGAAAACGAAAGGACACAGAGGAAGTCATACGATGAATGGACATTAGggtaccaatcaacctctggattacaaaatt |
| YU-2 | cattcacaggtgcagctcgaggggtcagggATGGAGAATAGATGGCAGGTTATGATAGTTTGGCAGGTAGATCGCATGAGAATTAGAGCTTGGAAGTCATTGGTGAAGCATCACATGTACATATCCGGGAAAGCGCGGGGTTGGTTTTATCGGCATCACTACGAATCACCACATCCTCGCATCAGCTCCGAAGTTCACATTCCTCTTGGGGATGCCAAACTTGTTATTACGACCTATTGGGGGCTGCATACTGGCGAGAGAGACTGGCATTTGGGTCAGGGTGTAAGTATAGAATGGCGCAAAAAGAGGTACTCAACCCAAGTCGATCCCGATTTGGCAGACCAACTGATCCATCTTTATTACTTTGACTGCTTCAGTGAGAGTGCGATACGGAAGGCGATTCTGGGATATCGAGTAAGTCCCCGCTGTGAATATCAAGCTGGTCATAATAAAGTGGGTTCTCTCCAATACCTGGCCCTTACGGCGTTGATAACTCCTAAAAAGACGAAACCTCCTCTTCCCTCCGTCAAGAAACTCACCGAAGACAGATGGAACAAGCCTCAGAAAACAAAGGGGCATAGAGGTTCCAGAACGATGAACGGACACTAGggtaccaatcaacctctggattacaaaatt |
| CH470 | cattcacaggtgcagctcgaggggtcagggATGGAGAACCGATGGCAGGTTATGATAGTCTGGCAAGTGGACAGGATGCGAATCAAAACGTGGAAGAGCTTGGTGAAACACCACATGCACATTTCCAAGAAGGCCCGCGGTTGGTTCTATCGCCACCATTACGAATCAACACACCCAAAGATCTCCTCAGAGGTCCATATCCCACTTCGAGAAGCACGGTTGGTAATAACAACGTACTGGGGGTTGCATACCGGCGAGAGGGATTGGCACTTGGGCCAGGGAGTATCCATTGAATGGAGAAAACGGAAGTACAGTACACAGGTGGACCCAGACCTCGCCGACCAATTGATTCACCTCTATTATTTCGACTGCTTCTCTGAGAGCGCGATTAGGAATGCGCTTCTGGGCCACATCGTGAGTCCGAGATGTGAGTACCAGGCCGGACATAACAAAGTGGGGAGCCTCCAGTATCTTGCCTTGACCGCTCTCGTTGCTCCAAAAAAGACGAAGCCTCCCCTGCCTAGCGTTAAAAAGCTGACCGAGGATAGGTGGAATAAGCCGCAAAAAACGAAGGGACACCGCGGGTCTCATACCATGTCTGGACACTAGggtaccaatcaacctctggattacaaaatt |

1. Vif open reading frames (ORFs) shaded in grey.

**Vif coding sequences in HIV-AFMACS viruses**

| **Virus** | **Sequence^1^** |
| --- | --- |
| ΔVpr-Vif_WT | atggaaaacagatggcaggtgatgattgtgtggcaagtagacaggatgaggattaacacatggaaaagattagtaaaacaccatatgtatATTtcaaggaaagctaaggactggttttatagacatcactatgaaagtactaatccaaaaataagttcagaagtacacatcccactaggggatgctaaattagtaataacaacatattggggtctgcatacaggagaaagagactggcatttgggtcagggagtctccatagaatggaggaaaaagagatatagcacacaagtagaccctgacctagcagaccaactaattcatctgcactattttgattgtttttcagaatctgctataagaaataccatattaggaTATAGAgttagtcctaggtgtgaatatcaagcaggacataacaaggtaggatctctacagtacttggcactagcagcattaataaaaccaaaacagataaagccacctttgcctagtgttaggaaactgacagaggac**agG**tggaacaagccccagaagaccaagggccacagagggagccatacaatgaatggacactag |
| ΔVpr-Vif_AYR | atggaaaacagatggcaggtgatgattgtgtggcaagtagacaggatgaggattaacacatggaaaagattagtaaaacaccatatgtatGCTtcaaggaaagctaaggactggttttatagacatcactatgaaagtactaatccaaaaataagttcagaagtacacatcccactaggggatgctaaattagtaataacaacatattggggtctgcatacaggagaaagagactggcatttgggtcagggagtctccatagaatggaggaaaaagagatatagcacacaagtagaccctgacctagcagaccaactaattcatctgcactattttgattgtttttcagaatctgctataagaaataccatattaggaGCTATAgttagtcctaggtgtgaatatcaagcaggacataacaaggtaggatctctacagtacttggcactagcagcattaataaaaccaaaacagataaagccacctttgcctagtgttaggaaactgacagaggac**agG**tggaacaagccccagaagaccaagggccacagagggagccatacaatgaatggacactag |
| ΔVpr-ΔVif | atggaaaacagatggcaggtgatgattgtgtggcaagtagacaggatgaggattaacacatggaaaagattagtaaaacaccatatgTAATAAtcaaggaaagctaaggactggttttatagacatcactatgaaagtactaatccaaaaataagttcagaagtacacatcccactaggggatgctaaattagtaataacaacatattggggtctgcatacaggagaaagagactggcatttgggtcagggagtctccatagaatggaggaaaaagagatatagcacacaagtagaccctgacctagcagaccaactaattcatctgcactattttgattgtttttcagaatctgctataagaaataccatattaggaGCTATAgttagtcctaggtgtgaatatcaagcaggacataacaaggtaggatctctacagtacttggcactagcagcattaataaaaccaaaacagataaagccacctttgcctagtgttaggaaactgacagaggac**agG**tggaacaagccccagaagaccaagggccacagagggagccatacaatgaatggacactag |

1. Based on native NL4-3 Vif sequence. Mutagenised codons shaded in grey. The mutation in codon 173 (highlighted in bold) is silent in Vif (AGA>AGG; both encoding Arg) but abrogates the Vpr start codon in the +2 reading frame (underlined).

**C-terminal 4xHA-tagged APOBEC3F coding sequence in pHRSIN-S-W-pGK puro**

| **Gene** | **Sequence^1^** |
| --- | --- |
| APOBEC3F | ATGAAGCCTCACTTCAGAAACACAGTGGAGCGAATGTATCGAGACACATTCTCCTACAACTTTTATAATAGACCCATCCTTTCTCGTCGGAATACCGTCTGGCTGTGCTACGAAGTGAAAACAAAGGGTCCCTCAAGGCCCCGTTTGGACGCAAAGATCTTTCGAGGCCAGGTGTATTCCCAGCCTGAGCACCACGCAGAAATGTGCTTCCTCTCTTGGTTCTGTGGCAACCAGCTGCCTGCTTACAAGTGTTTCCAGATCACCTGGTTTGTATCCTGGACCCCCTGCCCGGACTGTGTGGCGAAGCTGGCCGAATTCCTGTCTGAGCACCCCAATGTCACCCTGACCATCTCCGCCGCCCGCCTCTACTACTACTGGGAAAGAGATTACCGAAGGGCGCTCTGCAGGCTGAGTCAGGCAGGGGCCCGTGTGAAGATTATGGACGATGAAGAATTTGCATACTGCTGGGAAAACTTTGTGTACAGTGAAGGTCAGCCATTCATGCCTTGGTACAAATTCGATGACAATTATGCATTCCTGCACCGCACGCTAAAGGAGATTCTCAGAAACCCGATGGAGGCAATGTATCCACACATATTCTACTTCCACTTTAAAAACCTACGCAAAGCCTATGGTCGGAACGAAAGCTGGCTGTGCTTCACCATGGAAGTTGTAAAGCACCACTCACCTATCTCCTGGAAGAGGGGCGTCTTCCGAAACCAGGTGGATCCTGAGACCCATTGTCATGCAGAAAGGTGCTTCCTCTCTTGGTTCTGTGACGACATACTGTCTCCTAACACAAACTACGAGGTCACCTGGTACACATCTTGGAGCCCTTGCCCAGAGTGTGCAGGGGAGGTGGCCGAGTTCCTGGCCAGGCACAGCAACGTGAATCTCACCATCTTCACCGCCCGCCTCTACTACTTCTGGGATACAGATTACCAGGAGGGGCTCCGCAGCCTGAGTCAGGAAGGGGCCTCCGTGGAGATCATGGGCTACAAAGATTTTAAATATTGTTGGGAAAACTTTGTGTACAATGATGATGAGCCATTCAAGCCTTGGAAAGGACTAAAATACAACTTTCTATTCCTGGACAGCAAGCTGCAGGAGATTCTCGAGGCGGCCGCGGGTTCTGGTTACCCCTACGATGTGCCAGACTACGCTGGTTCTGGTTACCCATACGATGTTCCTGACTATGCGGGCTATCCCTATGACGTCCCGGACTATGCAGGTTCCTATCCATATGACGTTCCAGATTACGCTTAA |

1. 4xHA tag shaded in grey.

**Target sequences for RNAi**

| **Panel** | **Target** | **Sequence^1^** |
| --- | --- | --- |
| 1 | PPP2R5A | UGAAUGAACUGGUUGAGUA |
|  | PPP2R5B | GAACAAUGAGUAUAUCCUA |
|  | PPP2R5C | GGAAGAUGAACCAACGUUA |
|  | PPP2R5D | GGAAGAUGAACCAACGUUA |
|  | PPP2R5E | GCACAGCUGGCAUAUUGUA |
|  | DPH7 | CCTGGATTGCTGCTTTCAATT |
|  | FMR1 | GAGAGTTCAAGGCAGCTTGCC |
| 2 | PPP2R5A | CTGAAGACTGTTCTGCACCGA |
|  | PPP2R5B | AGTGTGTGGGGAGCACCCGGG |
|  | PPP2R5C | GAATGTGATCACAGAGCCTAT |
|  | PPP2R5D | AGCTGCTTGGCCACATCTCCA |
|  | PPP2R5E | CAATAAGCAGAGGCTGTTTGA |
|  | DPH7 | CACCCTGTCATGAATGCAGAG |
|  | FMR1 | CATGAACAGTTTATCGTAAGA |

**PCR primers for real time PCR**

| **Target** | **Primer** | **Sequence** |
| --- | --- | --- |
| PPP2R5A | PPP2R5A_Fwd | TTGGCCTCACATACAGTTGG |
|  | PPP2R5A_Rvs | CACGTTCTCTGGGATCTTCAC |
| PPP2R5B | PPP2R5B_Fwd | ACCAAACCATCGTATCACTGATC |
|  | PPP2R5B_Rvs | GACCTTGCCATAACTCCTGAC |
| PPP2R5C | PPP2R5C_Fwd | GTGAAGATCATGGAACCCCTC |
|  | PPP2R5C_Rvs | GCTGCGTTGTCACTGATTAAAC |
| PPP2R5D | PPP2R5D_Fwd | CCTTGAGTCTCCTGATTTCCAG |
|  | PPP2R5D_Rvs | AAAACTTGCCATAGATGCGATG |
| PPP2R5E | PPP2R5E_Fwd | TCACAGTTTAGGTCTCAAGGC |
|  | PPP2R5E_Rvs | ATCAGATAGCGTGTCCATGAAG |
| TBP | TBP_Fwd | GAGAAGATGGATGTTGAGTTG |
|  | TBP_Rvs | GATAGCAGCACGGTATGA |
